# The combined effect of SNP-marker and phenotype attributes in genome-wide association studies

**DOI:** 10.1111/j.1365-2052.2008.01816.x

**Published:** 2008-04

**Authors:** E K F Chan, R Hawken, A Reverter

**Affiliations:** Cooperative Research Centre for Beef Genetic Technologies, CSIRO Livestock Industries, Queensland Bioscience Precinct306 Carmody Road, St Lucia, Qld 4067, Australia

**Keywords:** genome-wide association studies, Hardy–Weinberg equilibrium, minor allele frequency, minor genotype frequency, quantitative traits, single nucleotide polymorphism, trait-distribution

## Abstract

The last decade has seen rapid improvements in high-throughput single nucleotide polymorphism (SNP) genotyping technologies that have consequently made genome-wide association studies (GWAS) possible. With tens to hundreds of thousands of SNP markers being tested simultaneously in GWAS, it is imperative to appropriately pre-process, or filter out, those SNPs that may lead to false associations. This paper explores the relationships between various SNP genotype and phenotype attributes and their effects on false associations. We show that (i) uniformly distributed ordinal data as well as binary data are more easily influenced, though not necessarily negatively, by differences in various SNP attributes compared with normally distributed data; (ii) filtering SNPs on minor allele frequency (MAF) and extent of Hardy–Weinberg equilibrium (HWE) deviation has little effect on the overall false positive rate; (iii) in some cases, filtering on MAF only serves to exclude SNPs from the analysis without reduction of the overall proportion of false associations; and (iv) HWE, MAF and heterozygosity are all dependent on minor genotype frequency, a newly proposed measure for genotype integrity.

## Introduction

Genome-wide association studies (GWAS) using single nucleotide polymorphism (SNP) markers have become increasingly popular for dissecting the genetics of complex traits (reviewed in Hirschhorn *et al.* 2002 and McCarthy *et al.* 2008). Therefore, it is invaluable to recognize and understand how confounding factors embedded within genotypic and/or phenotypic data may lead to spurious associations. This is particularly important in GWAS because associations are tested at tens to hundreds of thousands of SNP markers, inflating the rate of false associations (type I error).

A filtering process, defined by a set of rules, is generally applied to remove markers from an analysis. The deduction of these rules may be arbitrary (e.g. Easton *et al.* 2007; Sladek *et al.* 2007) or empirical (The Wellcome Trust Case Control Consortium 2007), and this is typically based on various measures or attributes calculated to reflect the markers’ integrity and usefulness. These attributes may include genotyping call-rate, missing data, monomorphism, loss of heterozygosity (LOH), observed heterozygosity (*H*_obs_), minor allele frequency (MAF), and extent of Hardy–Weinberg equilibrium (HWE) deviations. In this paper, we also propose minor genotype frequency (MGF) as a filtering criterion and explore its value as a quality control measure.

Call-rate and missing data can be used as an indicator of genotyping error and they remain the most commonly used measures of genotyping integrity (Di *et al.* 2005; Shen *et al.* 2005; Moorhead *et al.* 2006; Easton *et al.* 2007; Sladek *et al.* 2007; Shifman *et al.* 2008). Monomorphic SNPs are uninformative in genetic association studies as there is no genotypic difference. LOH (*H*_obs_ = 0) SNPs may impact on statistical power because of loss of information. SNPs with excessively high *H*_obs_ may reflect contamination and poor genotyping integrity (Teo *et al.* 2007). SNPs with low MAF have a frequency imbalance between the two allelic groups, which may in fact reflect functional importance (Cargill *et al.* 1999). SNPs deviating from HWE may confound trait-allele association as they are thought to reflect genotyping error (Clayton *et al.* 2005; Salanti *et al.* 2005), although the contrary has also been argued (Cox & Kraft 2006). Together, these warrant the need to understand the cost and benefits of filtering SNPs based on these properties.

To date, little research has been conducted using genome-wide SNP genotyping in cattle (e.g. Barendse *et al.* 2007; Hayes *et al.* 2007; Khatkar *et al.* 2007; Hayes & Goddard 2008), and only one group (Barendse *et al.* 2007) has reported a GWAS using cattle. Further, the majority of GWAS have adopted a case–control design whereby the traits of interest are binary (McCarthy *et al.* 2008). Appreciating many complex traits are continuous or ordinal, and recognizing the growing attention on these traits (e.g. Scuteri *et al.* 2007; Weedon *et al.* 2008), we also focus on the effects of trait properties on GWAS. We first introduce and report on the SNP attributes of an empirical data, then we proceed to examine the combined effects of various genotype and phenotype properties on false associations in GWAS.

## Materials and methods

### Samples and SNP genotype data

Five hundred and sixty-five Brahman cows were genotyped at 9075 SNPs using the MegAllele™ Genotyping Bovine 10k SNP Panel (Hardenbol *et al.* 2005). Genotyping calls were made, as part of Affymetrix's genotyping service, using TrueCall™ Analyzer (ParAllele BioScience; Moorhead *et al.* 2006).

Partial or full parentage for 486 animals was known. They were sired by 55 bulls averaging 10 (±7.6 SD) progenies/bull (max 47 progenies/bull) and 478 dams averaging one (±0.2 SD) progeny/dam (max three progenies/dam). Kinship coefficients were estimated using pedigree information of 9082 animals spanning up to seven generations and the parente program of the pedig package (Boichard 2002).

### Simulated phenotype data

Five trait-types were simulated according to the following distributions reflecting the majority of real data structures:

1. Continuous data with normal distribution, Normal(μ = 0, σ^2^ = 1).
2. Ordered categorical data with normal distribution,Binomial (*n* = 10, *P* = 0.5).
3. Ordered categorical data with discrete distribution,Binomial (*n* = 10, *P* = *X*), where *X*∼ Uniform (*a* = 0,*b* = 1).
4. Ordered categorical data with uniform distribution,Uniform (*a* = 0, *b* = 1).
5. Binary data with binomial distribution, Binomial(*n* = 1, *P* = 0.5).

For each trait-type, 1000 simulations were generated under the null hypothesis of no association where in each simulation, 565 random deviates were generated from the corresponding distribution.

### Test for Hardy–Weinberg equilibrium

Deviation from HWE was assessed using the chi-squared goodness-of-fit test and Fisher’s Exact test on the null hypothesis that *p*^*2*^*+*2*pq + q*^*2*^ = 1, where *p* and *q* are the two allelic frequencies (Emigh 1980). *P*-values for the two tests were obtained from the chi-squared (1 d.f.) and hypergeometric distributions respectively as per the *pchisq*() and *fisher.test*() functions in r/stats (R Development Core Team 2007).

### Genome-wide association test

Association between each trait at each polymorphic SNP was assessed using linear regression, where the simulated trait values across the 565 individuals were regressed onto the numeric code of each SNP genotype (i.e. 0, 1, or 2 copies of the alleles); this tested the null hypothesis of the additive allelic effect on the trait. Regression analyses were performed using *lm*() and *P*-values obtained from the *F*-distribution using *pf*() in r/stats. Significant associations were defined at point-wise *P* < 0.001 to ensure an average of one significant (and spurious) association per SNP across the 1000 replicates.

### Test for uniform distribution of *P*-values

To test whether association is independent of SNP attributes, we compared, using the Kolmogorov–Smirnov (KS) test, the observed distribution of the 8623 *P*-values (one from each polymorphic SNP) against the null distribution (a uniform distribution in the [0, 1] interval). *P*-values were obtained using the *ks.test*() function in r/stats. The median *P*-values from the 1000 KS tests were 0.14 ± 0.30 SD for continuous normal traits, 0.12 ± 0.24 SD for categorical normal traits, 0.12 ± 0.27 SD for categorical discrete traits, 0.12 ± 0.27 SD for categorical uniform traits and 0.02 ± 0.14 SD for binary traits.

### Correlation tests

To ascertain the relationship between a SNP attribute and the number of false positives (FPs), Spearman’s correlation coefficients (ρ) were calculated. Significant correlation was only asserted if |ρ| ≥ 0.1 at *P* < 0.05 (two-sided test against the null that ρ = 0). As per the *cor.test*() function in r/stats, *P*-values were computed using the AS 89 algorithm.

For each trait-type, we tested the null hypothesis that the numbers of SNPs across eight FP bins (FP = 0, 1, 2, 3, 4, 5, 6−10, >10) are the same between the ‘good’ and ‘bad’ SNP sets. Pearson’s chi-squared test was used for this purpose, with *P*-values obtained from 10 000 permutations using *chisq.test*() in r/stats.

Two tests were used for comparing the distributions of the same SNP attribute between FP-free (FP = 0) and FP-prone (FP ≥ 4) SNPs: (i) Pearson’s chi-squared test for LOH; and (ii) Mann–Whitney test for all other (non-binary) SNP attributes. *P*-values for the chi-squared test were determined from 10 000 simulations using *chisq.test*() and those for the Mann–Whitney tests were approximated from a Gaussian distribution using *wilcox.test*() in r/stats.

## Results

### SNP attributes

Each SNP has a median call-rate of 99.8% (85–100%), a median of one (range: 0–90) missing genotype, and 5% of SNPs are monomorphic. Excluding monomorphic SNPs, *H*_obs_ = 0.21 ± 0.17, of which 0.4% (33/8623) have LOH (*H*_obs_ = 0).

**Figure 1:**
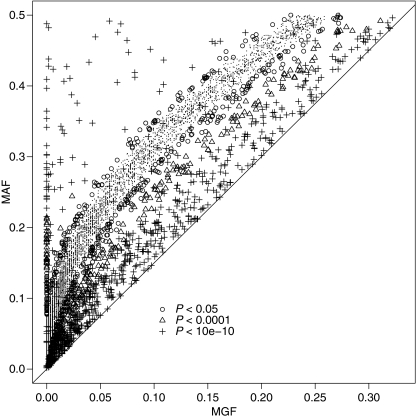
Relationship between minor genotype frequency (MGF) and minor allele frequency (MAF) for 9075 SNPs from 565 individuals. SNPs deviating from HWE at *P* < 0.05 (circle), *P* < 0.0001 (triangle) and *P* < 10 x 10^-10^ (cross) are indicated.

In this paper, we introduce and examine the effects of MGF on GWAS. The necessity to include MGF in addition to MAF is justified because SNPs with low MGF do not always imply low MAF (Fig. 1). An extreme example is LOH; of the 33 LOH SNPs, two have MAF > 0.4, suggesting equal selection pressure on the two homozygotes. Furthermore, the inclusion of MGF in addition to the test of HWE is because SNPs with low MGF do not necessarily deviate from HWE, as in the case when the minor genotype is one of the homozygotes. Of the 638 SNPs with 0 < MGF < 0.002 (averaging only one individual harbouring the minor genotype), 507 (79.5%) are in HWE.

Minor allele frequency is 0.10 ± 0.14 SD across all SNPs and MGF is 0.05 ± 0.07 SD, with the former figure increasing to 0.16 ± 0.14 SD following the exclusion of monomorphic markers, whilst the latter figure for MGF remains unchanged. Depending on the test statistic and associated criteria, between 13.6% [Fisher’s Exact test at *P* < 0.0001 for autosomal SNPs with MAF ≥ 0.05 as in Khatkar *et al.* (2007)] and 23.6% [Pearson’s chi-squared test at *P* < 0.05 for autosomal SNPs with at least five expected samples per genotypic group as in Barendse *et al.* (2007)], SNPs deviate from HWE. Our notably left-skewed MAF distribution [relative to that reported in Barendse *et al.* (2007)] and large numbers of HWE deviations are attributed to the elevated shared ancestry within our samples: average kinship coefficient is 0.020 ± 0.024 SD. In this paper, we use this to our advantage to explore the effect of HWE deviation on the extent of type I errors.

### Effects of SNP and phenotypic attributes on GWAS

We examined the effects of SNP attributes on type I errors in GWAS in consideration of five types of phenotypic traits. As we are interested in the extent of false associations, we chose to simulate these traits under the null hypothesis of no association: traits were purely simulated under the specified distribution independent of the animals and their genotypes, i.e. no genetic structure was simulated.

### Extent of false associations

Under our null hypothesis, two observations are expected: (i) *P*-value distributions should be uniform for each GWAS (i.e. each simulated trait); and (ii) an average of one FP should be observed for each SNP. Here, FP is the number of 1000 simulated traits passing the significance threshold of *P* < 0.001, and thus each SNP is expected to falsely associate with one of the 1000 simulated traits by chance alone (FP = 1).

**Figure 2:**
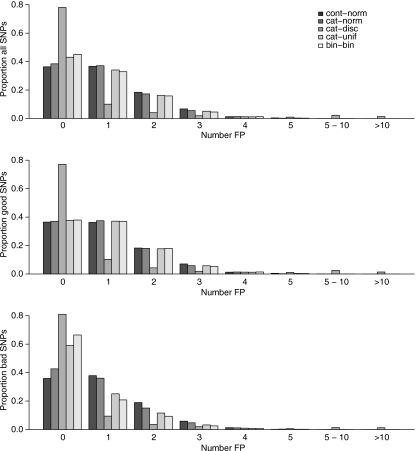
Proportions of SNPs with the corresponding number of false associations for the five trait-types. Shown are the proportions of all SNPs (top), ‘good’ SNPs (middle) and ‘bad’ SNPs (bottom). The five types of quantitative traits are: normally distributed continuous data (cont-norm), normally distributed ordered-categorical data (cat-norm), discretely distributed ordered-categorical data (cat-disc), uniformly distributed ordered-categorical data (cat-unif) and binomially distributed binary data (bin-bin).

The first expectation is satisfied by four trait-types; only simulated binary traits have *P*-values that are significantly non-uniform (median *P* = 0.02 for tests of uniformity), signifying an increased sensitivity of binary traits to various SNP attributes. The second expectation is satisfied by all but categorical-discrete traits (Fig. 2, top panel); instead of the majority of SNPs having FP = 1, only 10% SNPs complied, while >78% show no significant association (FP = 0).

### What SNP properties affect FP?

To identify SNP attributes that may influence false associations, we assessed the level of correlations between FP and each SNP attribute. Here, significant correlation is only asserted if |ρ| ≥ 0.1 and corresponding *P* < 0.01. Results show only significant correlations for categorical-uniform and binary traits (Table 1).

**Table 1:** Spearman’s ρ-correlation between number of false associations and various SNP attributes.

SNP attributes	Continuous normal	Categorical normal	Categorical discrete	Categorical uniform	Binary
Call-rate	–	–	–	–	–
Missing values	–	–	–	–	–
LOH	–	–	|ρ| < 0.1 (*P* = 0.009)	–	–
*H*_obs_	–	|ρ| < 0.1 (*P* < 10^−7^)	|ρ| < 0.1 (*P* < 10^−4^)	**ρ = 0.20 (*P* < 10**^−**16**^**)**	**ρ = 0.29 (*P* < 10**^−**16**^**)**
MAF	–	|ρ| < 0.1 (*P* < 10^−7^)	|ρ| < 0.1 (*P* < 10^−4^)	**ρ = 0.20 (*P* < 10**^−**16**^**)**	**ρ = 0.28 (*P* < 10**^−**16**^**)**
MGF	–	|ρ| < 0.1 (*P* < 10^−6^)	|ρ| < 0.1 (*P* < 10^−3^)	**ρ = 0.16 (*P* < 10**^−**16**^**)**	**ρ = 0.23 (*P* < 10**^−**16**^**)**
HWE: χ^2^-statistic	–	|ρ| < 0.1 (*P* < 10^−4^)	|ρ| < 0.1 (*P* = 0.017)	**ρ = 0.11 (*P*< 10**^−**16**^**)**	**ρ = 0.12 (*P* < 10**^−**16**^**)**
HWE: Fisher’s odds ratio	–	–	–	–	|ρ| < 0.1 (*P* < 10^−4^)

Only correlations where either |ρ| ≥ 0.1 or the corresponding *P* < 0.05 are shown, otherwise ‘–’ is indicated, and only when both criteria are satisfied is significance asserted (**bold**). For test of HWE, the chi-squared test was used for all SNPs, and Fisher’s Exact test was used only on SNPs with *n* ≥ 5.

The extent of false associations is not affected by call-rate, missing data, or LOH. It is, however, significantly affected by *H*_obs_ for categorical-uniform (ρ ≈ 0.2) and binary (ρ ≈ 0.3) traits. Because of the relationships between MAF, MGF and *H*_obs_(MAF = *x*+ ½*H*_obs_, where 0 ≤ *x*≤ 1; MAF ≥ MGF × 1.5), FPs are also significantly influenced by MAF and MGF with 0.16 ≤ ρ ≤ 0.28 for categorical-uniform and binary traits.

### Can filtering of SNPs reduce the extent of FPs?

Significant correlations between FP and various SNP attributes suggest that FP should decrease if problematic or ‘bad’ SNPs are eliminated prior to association. Here we assess this by comparing the extents of FPs from ‘good’ and ‘bad’ SNPs. As our objective was to investigate the impact of various SNP attributes on false associations, our null hypothesis here was that the extent of FPs are equal between the set of ‘good’ and ‘bad’ SNPs. In GWAS, SNPs are commonly excluded based on several criteria that generally reflect their informativeness and level of variation. These criteria are variable in the literature, and for the purpose of this study, ‘good’ SNPs are defined as those passing the following set of criteria derived from recent literature:

1. Call-rate ≥ 95% (e.g. Easton *et al.* 2007; Sladek *et al.* 2007; Shifman *et al.* 2008).
2. MAF ≥ 0.01 (e.g. Sladek *et al.* 2007).
3. HWE *P* ≥ 0.001 (e.g. Cupples *et al.* 2007; Sladek *et al.* 2007; Shifman *et al.* 2008).

These criteria classified 25% of polymorphic SNPs as ‘bad’ and 75% as ‘good’.

The extent of false associations between ‘good’ and ‘bad’ SNPs is not significantly different (*P* > 0.05; Fig. 2) for continuous-normal traits. Conversely, and paradoxically, the proportion of ‘good’ SNPs with FP = 0 is lower compared with that of ‘bad’ SNPs (Fig. 2, bottom two panels) for the remaining four trait-types, suggesting ‘bad’ SNPs are *less* vulnerable to spurious associations. This phenomenon extends to FP > 0; there is significant difference in the proportion of ‘good’ and ‘bad’ SNPs across the eight FP bins (*P* < 0.01) for all but continuous-normal traits. In particular, >59% of ‘bad’ SNPs have FP = 0 for categorical-uniform traits and <40% of the ‘good’ SNPs have FP = 0 for binary traits.

Retrospectively, these results are unsurprising, as ‘bad’ SNPs are less informative than ‘good’ SNPs and so should be more likely to incur *false negatives*. Yet, as our interest is in *false positives*, these results raise the question of which of the SNP attributes, if any, can ‘protect’ against FP. To address this, we compared various attributes of SNPs that are FP-free (FP = 0) and FP-prone (FP ≥ 4) and found (Table 2): (i) FP-prone SNPs have significantly higher frequencies of heterozygotes compared with FP-free SNPs in non-normally distributed traits; (ii) FP-prone SNPs have significantly higher MAF and MGF for all but continuous-normal traits; and (iii) many more FP-free SNPs have MGF = 0 (35–58%) compared with FP-prone SNPs (10–19%). These observations suggest low *H*_obs_, MAF, or MGF can limit false associations, particularly for ordinal and binary traits.

**Table 2:** The significance of testing the null hypothesis of no difference between FP-free (FP = 0) and FP-prone (FP ≥ 4) SNPs.

FP = 0 vs. FP ≥ 4	Continuous normal	Categorical normal	Categorical discrete	Categorical uniform	Binary
Call-rate	–	–	–	–	–
No. missing	–	–	–	–	–
LOH	–	–	–	–	–
*H*_obs_	–	–	<10^−3^ (higher)	<10^−9^ (higher)	<10^−16^ (higher)
MAF	–	0.023 (higher)	<10^−3^ (higher)	<10^−10^ (higher)	<10^−16^ (higher)
MGF	–	–	0.001 (higher)	<10^−8^ (higher)	<10^−13^ (higher)
MGF = 0	–	0.001 (lower)	<10^−3^ (lower)	<10^−4^ (lower)	<10^−4^ (lower)
HWE: χ^2^-test	–	0.007 (higher)	0.008 (higher)	0.017 (higher)	0.009 (higher)
HWE: Fisher’s Exact test	–	–	–	–	–

Only significant differences (*P* < 0.05) are shown, otherwise, ‘–’ is indicated. ‘Higher’ and ‘lower’ in parentheses indicate if the distributions are right or left shifted respectively in FP-prone compared with FP-free SNPs. For the test of HWE, the chi-squared test was used for all SNPs, and Fisher’s Exact test was used only on SNPs with *n* ≥ 5.

False positive-prone SNPs deviate from HWE more often than FP-free SNPs, but this difference disappears when 49% SNPs with at least one of the three genotypes represented by less than five (or <1%) individuals are excluded. We infer from this that deviation from HWE alone does not affect false associations, rather FP is dependent on low MGF-induced HWE deviation. Again, continuous-normal traits appear unaffected by this.

### Trade-off between reduction in false positives and loss of SNPs

Finally, we explored the trade-off between the number of FP we can reduce and the number of useful SNPs we can retain. In particular, we examined the effects of all three-way combinations of MAF, MGF and HWE threshold at:

1. MGF ≥ 0, MGF > 0, MGF > 0.005, MGF > 0.01, MGF > 0.05, MGF > 0.1.
2. MAF > 0, MAF > 0.005, MAF > 0.01, MAF > 0.05, MAF > 0.1.
3. HWE: *P*≥ 0, *P*> 10^−6^, *P* > 10^−5^, *P* > 10^−4^, *P* > 10^−3^, *P* > 10^−2^, *P* > 0.05.

**Figure 3:**
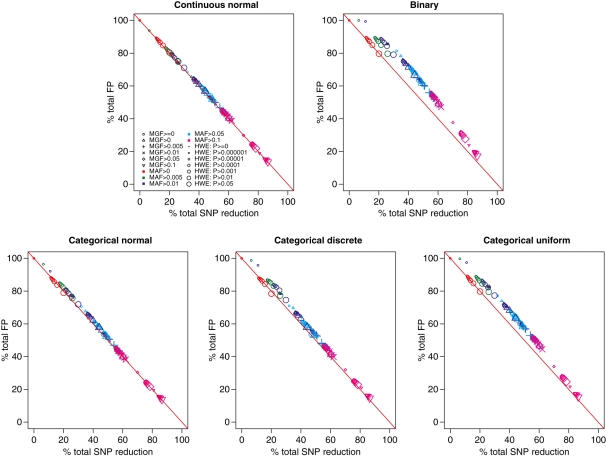
Rates of reduction in the proportion of false associations to the proportion of excluded SNPs at various combinations of MAF, MGF and HWE deviation thresholds. Filtration on MGF is indicated by different plotting symbols (circle: no filtration on MGF, triangle: MGF > 0, plus: MGF > 0.005, cross: MGF > 0.01, diamond: MGF > 0.05, inverse triangle: MGF > 0.01), filtration on MAF is indicated by different colours [red: polymorphic (MAF > 0), green: MAF > 0.005, blue: MAF > 0.01, cyan: MAF > 0.05, magenta: MAF > 0.01] and filtration on HWE deviation is indicated by different plotting sizes (smallest: no filtration on HWE deviation, to the largest: *P* > 0.05). The red line indicates the line of unity.

For most traits, the rate of FP reduction is proportional to the rate of SNP loss (Fig. 3), i.e. removing *x*% of the SNPs removes ∼*x*% of FP. This is particularly true for continuous-normal traits, reaffirming that the loss (and gain) of FP is random and thus proportional to the number of SNPs excluded from analysis.

However, for binary, categorical-discrete and categorical-uniform traits, some combinations of SNP filtration criteria result in more rapid SNP loss than FP loss. Specifically, an increase in MAF stringency only serves to increase the number of excluded SNPs but does not reduce the extent of false associations. (In Fig. 3, there is a shift of data-points above line of negative unity with increasing MAF stringency.) And finally, we show that the reduction in SNPs (and FPs) is more rapid from no filtration on MGF (circle) to MGF ≤ 0.05 (upside-down triangle) compared with no filtration on HWE deviation (smallest circle) to deviation at *P* ≤ 0.05 (largest circle).

## Discussion

Association studies are based on the fundamental assumption that the genetic variants underlying a phenotypic trait will co-segregate with the trait of interest in a given population. The statistical analyses are thus aimed at identifying the markers whose genotypes correlate best with the trait values across a population of individuals. Clearly, factors affecting the characteristics of either or both the phenotypic or genotypic data can severely affect the power and accuracy of detection.

In this paper, we have shown that some, but not all, of the examined SNP-attributes can influence spurious associations, and that the effect is not always negative and certainly not applicable to all trait-types. In particular, none of the SNP attributes appear to have major effects on normally distributed traits, be it continuous or ordered-categorical (Table 1). Only when we compare attributes of FP-prone and FP-free SNPs do we notice the effects of several SNP attributes on false associations of the latter trait-type (Table 2).

One such attribute is MGF. The influence of zero or near-zero MGF is not limited to categorical-normal traits and its effect is, surprisingly, not negative with respect to type I error. We have shown repeatedly that SNPs with low MGF tend to have fewer false associations across all trait-types. Ironically, this is a consequence of reduced statistical power in association tests, which would normally prevent, or reduce true as well as false associations. Thus, although we have shown that SNPs with zero or near-zero MGF tend to protect against false associations, we suspect it would conversely inflate false negatives (type II error).

In addition (and in some cases as a consequence of) low to zero MGF, low MAF, low *H*_obs_ and deviation from HWE can also protect against false associations; this is especially true for categorical-uniform and binary traits. Again, this is because SNPs with these attributes are susceptible to false negatives. In the case of deviation from HWE, and possibly for low *H*_obs_ and MAF, its effect is only manifested when the corresponding SNP also has near zero MGF. In fact, we failed to establish any connection between deviation from HWE and false associations with any trait-type for SNPs with MGF < 0.009 (corresponding to fewer than five individuals per genotype). This finding is of particular importance in GWAS, because deviation from HWE is a widely used SNP quality control measure.

While HWE deviation-induced FP for binary traits have been noted previously (Schaid & Jacobsen 1999), we have further demonstrated that the effect extends to categorical-uniform traits and that the effect is likely restricted to low MGF-induced HWE deviation. Moreover, while LOH (*H*_obs_ = 0) markers (with sufficiently low MAF to escape detection from HWE deviation) have been shown to cause false associations in transmission-disequilibrium tests (Hirschhorn & Daly 2005), here we demonstrated that the effect of near-zero *H*_obs_ is only a subclass of the larger problem of near-zero MGF in GWAS. For this reason, we strongly advise that deviation from HWE be used with caution or in conjunction with MGF as an inclusion/exclusion measure for genetic association studies.

To allow for easy comparison of the effects of genotype attributes on different trait-types, we have chosen to use a linear regression model for test of association for all trait-types. This is generally acceptable for quantitative traits, which are either normal or can be transformed to normality (e.g. Scuteri *et al.* 2007). However, this is not applicable to truly non-normal data. For this reason, such data types can be more prone to type I errors. We have shown this to be particularly true for binary and uniformly distributed ordinal traits, because of the relative increased probability of sampling from the tails of these distributions. For binary traits, alternate association test methods such as logistic regression (e.g. The Wellcome Trust Case Control Consortium 2007) and the Cochran–Armitage test (e.g. Fellay *et al.* 2007) are well-developed and commonly adopted. Conversely, there is little research into more appropriate methods for analysing ordinal and non-normally distributed traits. With the increasing popularity of GWAS, perhaps it is time for the community to direct more attention to this area.

Finally, two technical points are of note here. First, although we recognize that the genotype data used in this study are from one cattle population with its inherent family structure, the relationship between SNP and phenotypic attributes and their effects on spurious genetic associations are population-independent and thus should be applicable to other (non-cattle) populations. For example, although this population demonstrated a relatively low MAF across all SNPs (32% polymorphic SNPs with MAF < 0.05), the only difference compared with a population with a higher average MAF is the extent of FP. The nature of the effect of low MAF and the fact that the effect would be more prominent for categorical-uniform and binary traits is indisputable. Clearly, in order to make inference on statistical power and type II error, one would have to model family structure into the phenotype data and then account for it in the association test (e.g. Marchini *et al.* 2004).

Secondly, several studies have claimed that genotyping error can confound association studies because of distortion of allele frequencies (e.g. Gomes *et al.* 1999; Hosking *et al.* 2004; Salanti *et al.* 2005). Although we did not find any effect of genotyping call-rate and genotyping failure (missing data) on GWAS, we acknowledge that these are not true measures of genotyping accuracy. These measures are highly dependent on the genotyping platform, corresponding genotype-calling algorithm and their inherent limitations (Hardenbol *et al.* 2005). Thus, it remains unclear whether a more accurate measure of genotyping call-rate that is more reflective of genotyping error would reveal significant impact on GWAS; again, further study is needed.

In conclusion, we emphasize that whether an SNP is FP-free or FP-prone is highly dependent on *H*_obs_, MAF and MGF, as well as the characteristic and distribution of the trait which the SNP is to be tested against. Furthermore, the fact that an SNP is FP-free does not necessarily imply that it will be more efficient in a test of association, because the FP-free nature may simply be a reflection of the SNP’s inherent lack of statistical power for such a purpose.
